# Where is the policy? A bibliometric analysis of the state of policy research on medical tourism

**DOI:** 10.1186/s41256-020-00147-2

**Published:** 2020-05-06

**Authors:** Altaf Virani, Adam M. Wellstead, Michael Howlett

**Affiliations:** 1 Lee Kuan Yew School of Public Policy, National University of Singapore, 469C Bukit Timah Road, Singapore, 259772 Singapore; 2 Department of Social Sciences, Michigan Technological University, Houghton, USA; 3 Department of Political Science, Simon Fraser University, Burnaby, British Columbia Canada

**Keywords:** Medical tourism, Health tourism, Healthcare tourism, Policy research, Policy thinking, Bibliometric analysis

## Abstract

**Background:**

It is imperative that researchers studying medical tourism connect their work with policy, so that its real-world challenges can be better understood, and more effectively addressed. This article gauges the scope and evolution of *policy thinking* in medical tourism research through a bibliometric review of published academic literature, to establish the extent to which researchers apply public policy theories and frameworks in their investigation of medical tourism, or consider the policy imperatives of their work.

**Methods:**

A Boolean search of the Web of Science (WoS) Core Collection was performed to identify policy-related publications on medical tourism. We analyzed the results using bibliometrics and a data visualization software called *VOSviewer* to identify patterns in knowledge production and underlying network linkages in policy research on the subject.

**Results:**

Our findings suggest that only a small proportion of medical tourism research explicitly addresses policy issues or applies policy paradigms in their study approach. Field-specialized journals serving practitioners publish less research as compared to interdisciplinary social and health policy journals. Moreover, there are significant geographical and disciplinary disparities in the policy-orientation of research, and a predilection towards select policy areas such as reproductive and transplant tourism to the neglect of more holistic governance and health system considerations.

**Conclusion:**

This article is a call to action for greater engagement by policy scholars on medical tourism, and for health researchers to more explicitly consider how their research might contribute to the understanding and resolution of contemporary policy challenges of medical tourism. Failure to clearly and consistently make the policy connection is a lost opportunity for researchers to frame the public debate, and influence policy thinking on medical tourism.

## Background

Even as policymakers around the world are focusing their attention on medical tourism as a welfare and developmental strategy, many have failed to articulate a clear and comprehensive policy vision. As a result, most initiatives in this sector operate in a policy void [1, 2]. A similar neglect of *policy thinking* is seen in research on medical tourism. Situated at the intersections of knowledge streams, medical tourism has attracted researchers from diverse fields, bringing methodological diversity and distinct disciplinary perspectives to its study [3]. Yet, this subject remains relatively untouched by public policy researchers. A significant amount of research on medical tourism, even if potentially policy-relevant, is undertaken and consumed by researchers outside the formal confines of public policy. As a result, there exists a yawning research-policy gap in the medical tourism field [4]. Such gaps have been observed in both scientific and policy literature on climate change adaptation, which is partly responsible for the disconnect between climate change research and policy [5, 6]. Lack of adequate engagement with policy-oriented research is a major issue because it limits our understanding of the diverse challenges of medical tourism in different sectors and across various countries, and the ability of policymakers to devise effective contextual strategies to address them. For instance, while a significant body of emerging research on medical tourism has shown how it can burden public resources and exacerbate health inequities, affecting the most vulnerable populations in destination countries [7–10], much of the policy continues to focus on measures for easing outbound medical travel from source countries, and creating supply-side capacities and competitiveness in destination countries, without sufficiently addressing the systematic drivers of medical tourism (e.g. health system deficiencies, lack of health coverage and regulatory barriers) or correcting its distortionary effects. The relative inability of medical tourism research to cast these issues in policy terms and suggest actionable solutions has contributed to the lop-sidedness in policy priorities, and to the shifting policy narrative on medical tourism from a vehicle for socioeconomic development, health sector improvement and universal health coverage, to one for economic growth and healthcare outsourcing [4]. In other instances, policy research has failed to find uptake due to political or other reasons. In the UK, for example, the political narrative driving policy against inbound medical tourism and healthcare coverage for migrants is unsupported by empirical evidence on the lack of systematic abuse of the NHS [11]. This suggests a pressing need to strengthen research-policy linkages in the sector. It is therefore important for researchers and policymakers to purposively consider and engage with the policy aspects of medical tourism so that its policy goals can be achieved, while its unintended adverse consequences can be mitigated. Public policy as an academic discipline can significantly enhance our understanding of medical tourism and provide critical insights to help address its challenges, making it imperative for medical tourism researchers to imbibe some of its objectives and approaches.

A policy perspective offers several advantages. Since no single discipline is independently equipped to understand the challenges of medical tourism, a macro framework is required to weave these perspectives into coherent knowledge, and to provide a common language for effective exchange between domain experts with sector-specific knowledge, and academic researchers whose work is primarily grounded in the scholarship of their respective disciplines. Such fissures have been observed, for instance, between normative and empirical research on ethical issues in medical tourism, resulting in poor cross-fertilization and engagement among research scholars with diverse orientations [12]. Being interdisciplinary, public policy is suitably positioned to connect diverse research agendas and methodologies that medical tourism research broadly encompasses.

Moreover, its action-orientated approach is germane to distill practical policy insights from theoretical research for consumption by policymakers. Empirically informed policy articles are typically problem-focused and attempt to situate their research in the context of contemporary policy challenges (e.g. health inequities, unethical practices, health workforce depletion, policy incoordination and regulatory mismatch). They can provide policymakers with practical insights on what works, what doesn’t and why, so they can design more effective regulatory interventions.

**Fig. 1:**
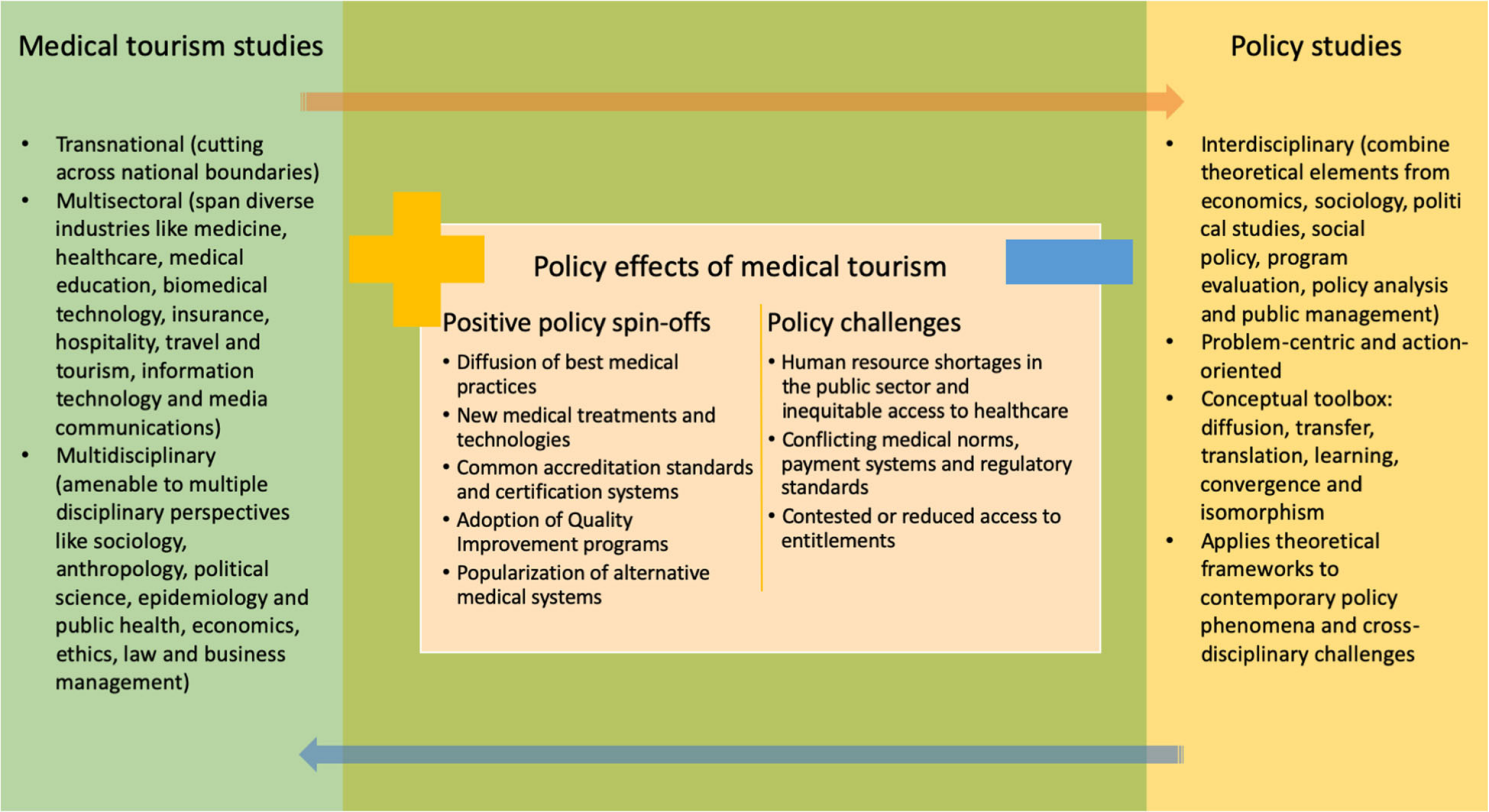
The intersection of medical tourism and policy studies

In addition to the inclusivity and instrumentality advantages, public policy offers distinctive conceptual tools that can help policy scientists analyze the dynamics of exchanges that constitute or are triggered by medical tourism. Hall and Jenkins [13] have alluded to the relative absence of theoretical frameworks in tourism studies that hinder systematic analysis of tourism policies. Policy scientists have routinely applied such mechanisms as *diffusion*, *transmission*, *transfer*, *translocation*, *translation*, *learning*, *emulation*, *adaptation*, *coalescence*, *maturation*, *hybridization*, *convergence*, *isomorphism*, *mutation* and *coercion* to study the dynamics of contemporary policy phenomena, and the nature and triggers of policy change [14–16]. With its mid-level theory focus, the policy sciences provide a range of empirically tested frameworks and theories that can help identify the causal triggers of medical tourism, evaluate its global health effects and formulate theory-driven responses to its challenges (Fig. 1).

There has been recent work on identifying thematic trends in medical tourism research [17, 18]. However, its engagement with policy is mostly unexplored. In this article, we examine the current state of medical tourism research to try and establish the extent to which researchers apply public policy theories and frameworks in their investigation of medical tourism or consider the policy imperatives of their work. We employ the PRISMA (Preferred reporting Items for Systematic review and Meta-Analyses) process flow developed by Moher, Liberati et al. [19] to identify and synthesize peer reviewed policy literature on medical tourism through an online database search. We map the size and evolution of scientific activity, patterns of knowledge transfer, and the nature of extant enquiry in policy research on medical tourism [20, 21]. Based on our review, we describe the state of policy research on medical tourism and suggest how scholars can further contribute to the understanding and resolution of contemporary policy challenges of medical tourism.

## Methods

**Fig. 2:**
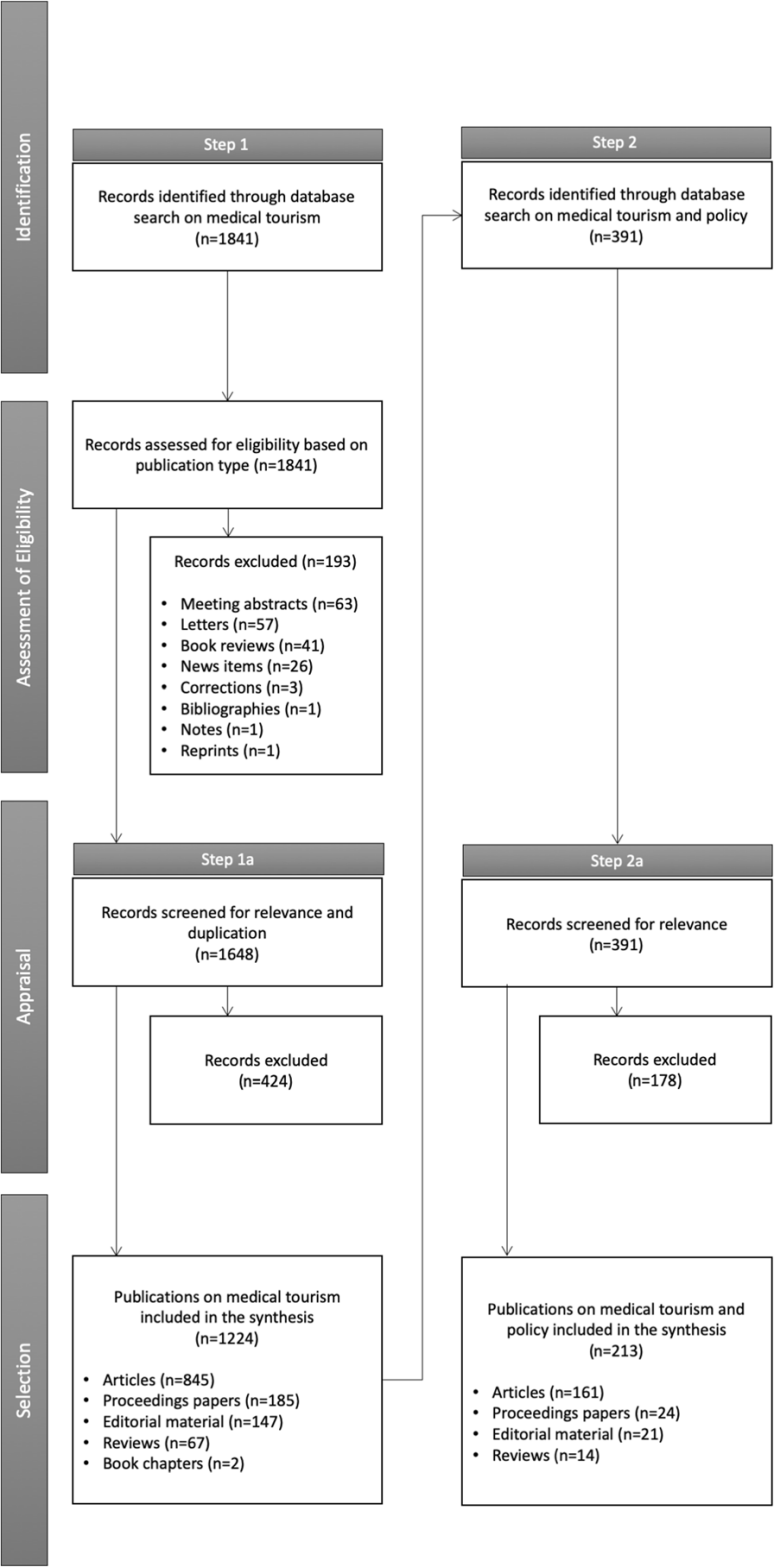
Steps in the review process

The process flow in Fig. 2 illustrates the different stages in our review process and the methods used for the search, screening and selection of studies for inclusion in the synthesis. A brief description of the methodology follows.

### Search strategy

We performed a search of the Web of Science (WoS) Core Collection between October and November 2018 to get the lay of the land of medical tourism literature. Given the multidisciplinary nature of our enquiry, the need for uniform indices and the technical challenges of multi-source comparative analyses, we limited our search to the WoS database, and did not include discipline-specific databases. Nonetheless, a significant proportion of their publications are cross-indexed in WoS, and therefore, while our search is not all-inclusive, it provides us with a reasonably representative snapshot of the state of medical tourism and policy research.

The initial search employed the following Boolean string to identify publications that refer to medical tourism in their titles, abstracts or keywords: “medical tour*” OR “health tour*” OR “healthcare tour*” OR “surgical tour*” OR “transplant tour*” OR “cosmetic tour*” OR “reproductive tour*” OR “abortion tour*” OR “wellness tour*” OR “medical travel” OR “health travel” OR “healthcare travel” OR “surgical travel” OR “cosmetic travel” OR “reproductive travel” OR “wellness travel” (step 1). The search was then repeated using the following query to identify a subset of publications that reference policy: (“medical tour*” OR “health tour*” OR “healthcare tour*” OR “surgical tour*” OR “transplant tour*” OR “cosmetic tour*” OR “reproductive tour*” OR “abortion tour*” OR “wellness tour*” OR “medical travel” OR “health travel” OR “healthcare travel” OR “surgical travel” OR “cosmetic travel” OR “reproductive travel” OR “wellness travel”) AND (“policy” OR “policies” OR “regulat*” OR “governance” OR “reform”) (step 2).

### Inclusion and exclusion

Publications referring to medical tourism and policy were sequentially shortlisted. We covered both open access and subscription based publications for all years till date including journal articles, reviews, books and book chapters, conference proceedings, editorial material, trade publications and industry reports, but excluded extraneous items like reprints, book reviews, news articles, letters, meeting abstracts, short surveys, conference reviews, errata, bibliographies and notes. No language or geographical restrictions were applied to search results.

### Appraisal

We refined search results through an appraisal of identified publications as sub steps. Based on a review of the publication titles and abstracts, only those that directly focused on medical tourism (step 1a) and explicitly considered its policy dimensions or applied public policy theories and frameworks in their analysis (step 2a) were included. Full texts were scrutinized if the titles and abstracts were either ambiguous or insufficient for making a determination. Those making only tangential references to medical tourism and policy were excluded. Publications alluding to undefined policy implications but not substantively engaging with policy substance were also omitted. Duplicate records were removed prior to extraction.

### Data extraction

Screened results were saved into separate marked lists at the end of each search iteration. We used the inbuilt tools for citation analysis within WoS to extract metrics, categorize and rank results and visualize outputs. The data files were cleaned and imported into a data visualization software called *VOSviewer* to create and visualize network maps based on collated bibliographic data [22].

### Analysis

The publications thus identified were categorized and reviewed across a range of variables. We examined the size, state and evolution of scientific activity in terms of the top publications and citation metrics, type of publications, top journals, research areas covered, countries or regions from where this research is emanating, and languages in which the research is being published, to identify patterns in the production of knowledge on medical tourism and policy.

We conducted a bibliometric review to identify the underlying network linkages in published literature using the *VOSviewer* software. Bibliometric techniques have been used to explore research trends in fields such as molecular research [23], transplant medicine [24], climate science [25] and even medical tourism [18]. Policy researchers have likewise used these methods to examine the literature on policy implementation [26] and policy learning [27], research in health policy [28] and the policy sciences [29, 30], and the evolution of science and technology policies [31].

We created network maps to analyse the connectedness of publications through assessments of the frequency of simultaneous citation (co-citation) and common keywords and shared content within publication titles and abstracts (co-occurrence). The full counting method was used for ease of interpretation and temporal stability [32, 33].

## Results

### Production trends in policy-oriented medical tourism research

Out of the 1841 records captured through the initial search, we identified 1224 publications (845 articles, 185 proceedings papers, 147 editorial materials, 67 reviews and 2 book chapters) focused on medical tourism. Only 213 of these (161 articles, 24 proceedings papers, 21 editorial materials and 14 reviews) explicitly address some policy issue, or apply public policy concepts or frameworks in their examination of medical tourism, which comprises just over a sixth of the research. The majority of researchers thus do not situate their work in the context of policy action or seek to directly engage with government policy, even if they extend our knowledge of the phenomenon and its effects in ways which might potentially have a bearing on policy.

**Fig. 3:**
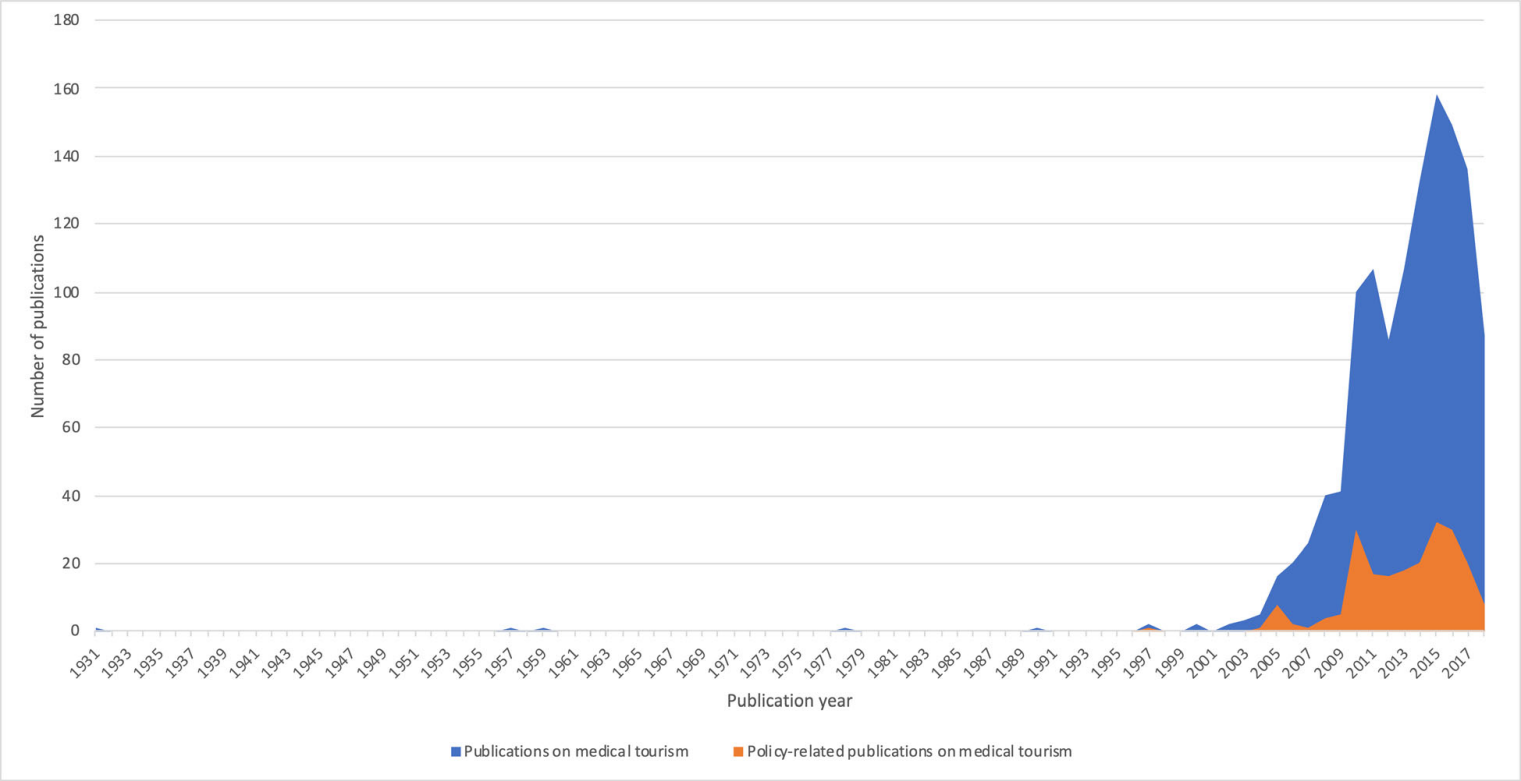
Publication trends in medical tourism and policy research

While the earliest known publication on medical tourism dates back to a 1931 article in the *British Medical Journal* on the impressions of medical tourists in Russia about Soviet medicine and hygiene [34], the first policy-related article appeared in *Health Policy* over six decades later reviewing the implementation of the European Union (EU) policy on mobility in the context of access to UK’s National Health Service (NHS) for EU visitors [35]. Research on medical tourism had been infrequent until that point. With growing focus on globalization, academic scholars and policymakers became more interested in understanding the nature of underlying processes, and their influence on international politics, national economies and welfare systems. This led to a spurt in research on medical tourism as well as its policy imperatives after 2000. Their growth trajectories have since continued, except that research on medical tourism overall is growing much faster than policy-related research on medical tourism (Fig. 3).

### Geographical distribution

The production of policy research in the field is geographically uneven, with some countries producing significantly more policy research than others. Much of it has originated in the western world. Nearly 60% is from authors based in the United States (US), United Kingdom (UK) and Canada (Table 1). What is surprising is that while the US is the unrivalled leader in terms of the quantum of policy research on the subject, it ranks far below major knowledge economies in terms of the proportion of policy-related work as a function of overall research output. Just over a sixth of medical tourism research that originates in the US addresses policy, which is about the average for the field. Australia fares only marginally better at about one fifth. In contrast, about a third of the research in the UK and Canada is policy-related (Table 2).

**Table 1:** Country affiliation of authors publishing most policy-related research on medical tourism (selected list)

Author country affiliation	Number of policy-related publications	Proportion of total policy research output (%)
US	50	23
UK	43	20
Canada	33	15
Australia	17	8
India	11	5
Netherlands	11	5

**Table 2:** Proportion of policy-related publications on medical tourism by authors in various countries (selected list of countries with ≥5 policy-related publications)

Author country affiliation	Number of publications on medical tourism	Number of policy-related publications	Proportion of publications that are policy-related (%)
Singapore	16	8	50
Czech Republic	11	5	45
UK	120	43	36
Brazil	14	5	36
Netherlands	33	11	33
Belgium	19	6	32
Canada	109	33	30
Switzerland	17	5	29
India	43	11	26
Iran	29	6	21
Australia	86	17	20
US	286	50	17
China	54	9	17
South Korea	49	7	14
Malaysia	64	7	11
Taiwan	45	5	11

Malaysia is the only middle-income economy with a significantly high production of medical tourism research, placing it in the same category as countries like the UK, Canada and Australia, but its contribution to policy research is negligible, with just over a tenth of its research focusing on policy. India and the Netherlands, on the other hand, are more modest producers of medical tourism research, but between a fourth and a third of their research is policy-related. Researchers in the city-state of Singapore, while making only a small contribution statistically, are consistently more policy-oriented, with half their publications explicitly addressing policy matters. This possibly reflects the strength of Singapore’s policy-focused departments, research centres and think-tanks, and its culture of research-driven governance and policymaking.

### Publication venues

Policy publications are concentrated in interdisciplinary health and social policy journals, while field-specialized journals like *Tourism Management*, the *Journal of Travel & Tourism Marketing*, the *International Journal of Healthcare Management*, the *Asia Pacific Journal of Tourism Research*, *Current Issues in Tourism* and *Transplantation* which publish a large volume of medical tourism research and cater to practitioners outside the traditional policy universe, publish significantly less policy work (Table 3). This is not surprising given that researchers target journals based on their editorial aims and readership, and journals vet manuscripts for alignment and relevance leading to self-selection, but it implies that the scholarship and readership in medical tourism research is fragmented between the *technical* and *applied* categories, with the latter relatively more policy-oriented. *Social Science & Medicine*, with an uncharacteristically low share of policy-related articles despite its high publication count on medical tourism, is a notable exception among social science journals. It is also remarkable that while a majority of publications in political science and public administration journals are policy-focused, these account for a fraction of the total volume of policy work, indicating a predominance of applied policy analysis in policy research on medical tourism, and inadequate theoretical engagement by public policy scholars with mainstream research.

**Table 3:** Policy-related publications on medical tourism in various journals (selected list)

Source title	Number of policy-related publications	Proportion of publications on medical tourism that are policy-related (%)
Globalization and Health	8	44
Global Social Policy	7	88
Reproductive Biomedicine Online	7	41
Frontiers in Public Health	5	100
Developing World Bioethics	4	50
Health Policy	4	57
International Journal of Health Services	4	50
Journal of Law and Medicine	4	80
American Journal of Bioethics	3	38
American Journal of Transplantation	3	38
BMC Health Services Research	3	30
Human Reproduction	3	50
International Journal for Equity in Health	3	50
International Journal of Feminist Approaches to Bioethics	3	38
International Journal of Health Policy and Management	3	50
Iranian Journal of Public Health	3	23
Medical Law Review	3	75
Social Science & Medicine	3	14
Tourism Management	3	10
Current Issues in Tourism	2	15
Journal of Travel & Tourism Marketing	2	10
Asia Pacific Journal of Tourism Research	1	7
Journal of Comparative Policy Analysis	1	100
Policy & Politics	1	100
Policy and Society	1	100
Politics & Policy	1	100
Social Policy & Administration	1	100

### Authorship

**Fig. 4:**
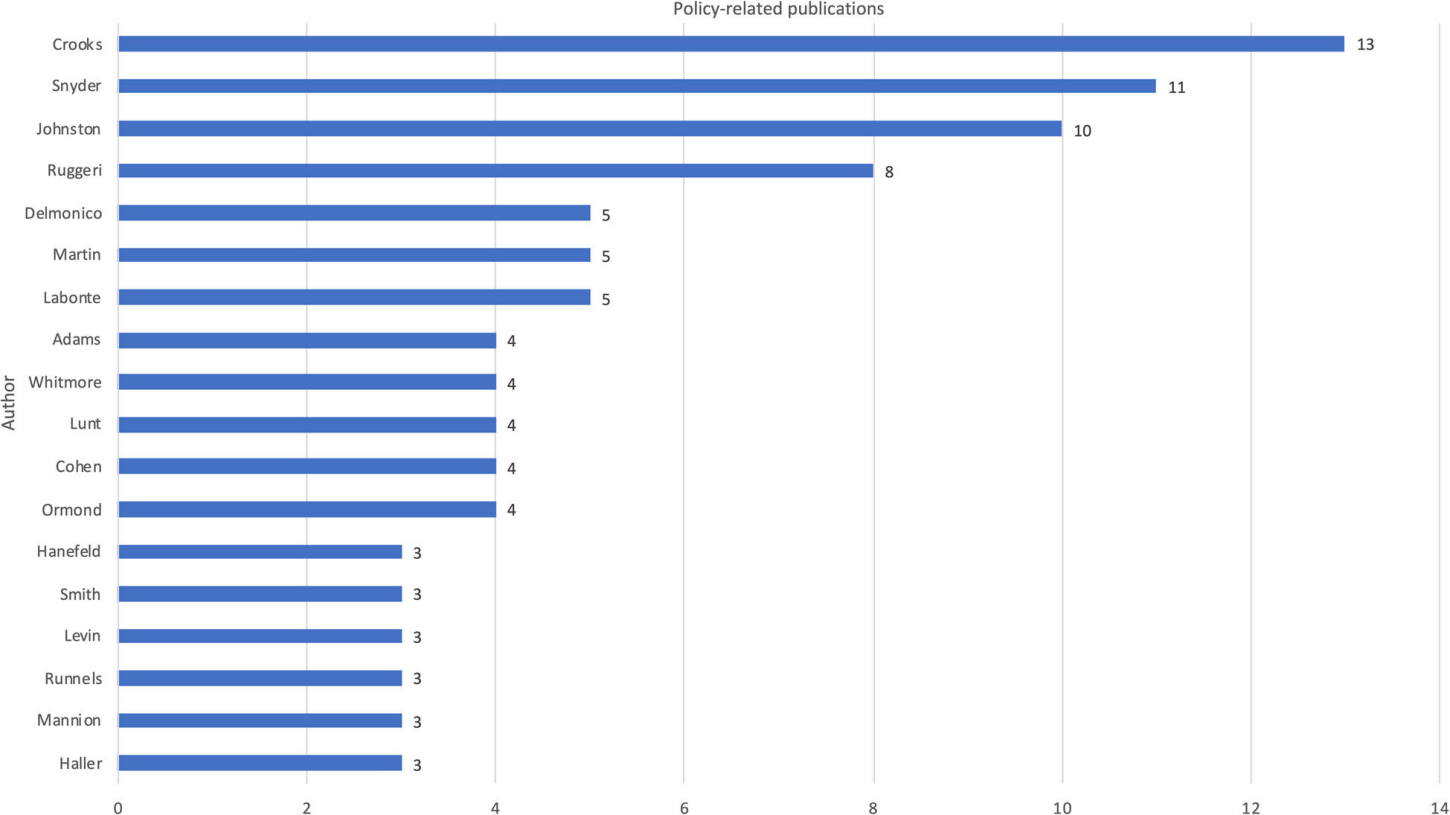
Authors producing most policy-related publications on medical tourism

Out of the 2611 authors who’ve published work on medical tourism, just over a fifth have engaged with policy. Of these, only 23 have three or more policy-related publications (Fig. 4). Crooks [13], Snyder [11] and Johnston [10] lead the pack, followed by Ruggeri [8], Delmonico, Martin and Labonte (5 each), Adams, Whitmore, Lunt, Cohen and Ormond (4 each), and Hanefeld, Smith, Levin, Runnels, Mannion, Haller, Tsai, Mainil, Blyth, Pennings and Whittaker (3 each).

**Fig. 5:**
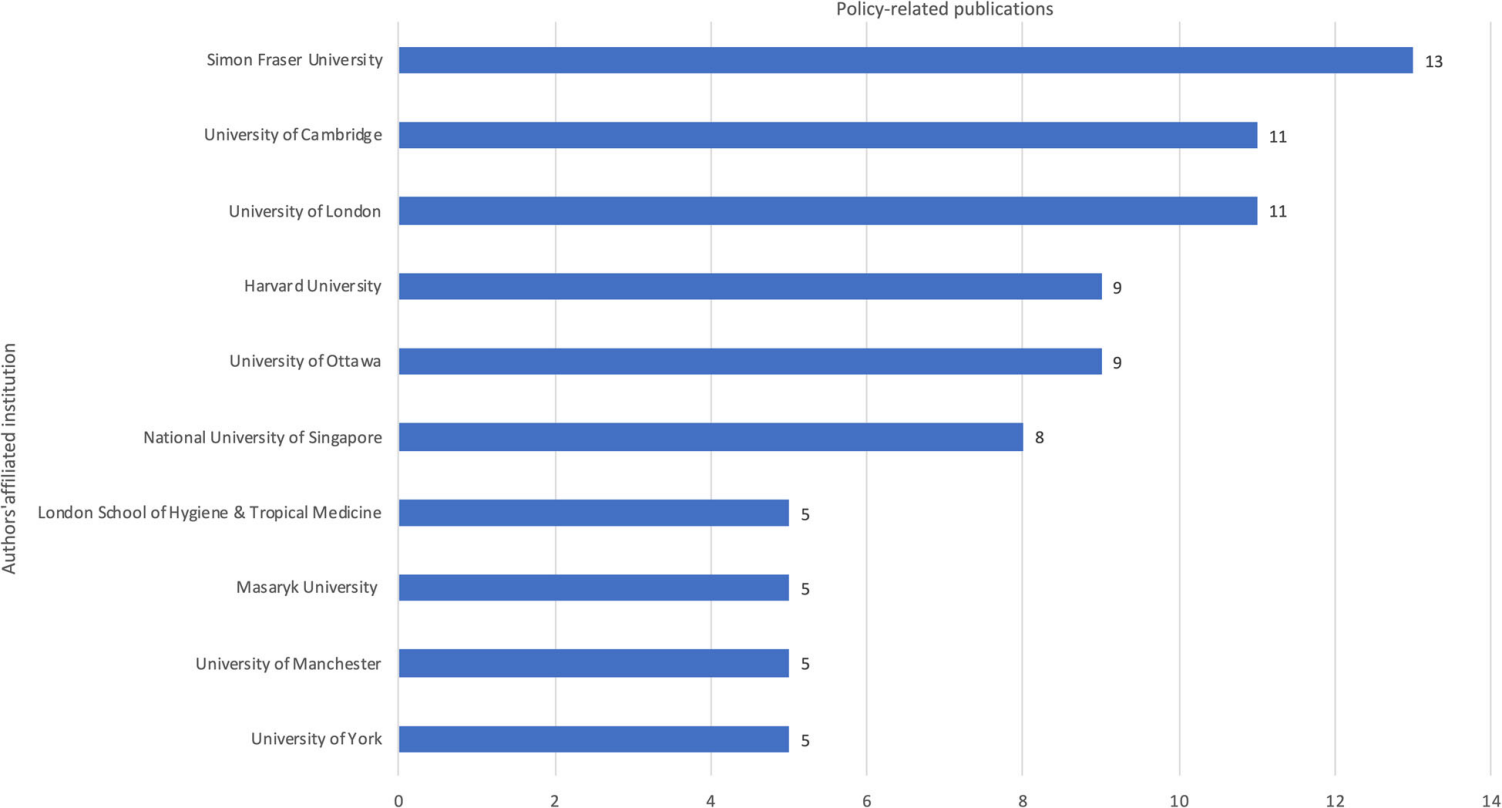
Institutional affiliations of authors producing most policy-related publications on medical tourism

A majority of these researchers are affiliated to only a handful of institutions. Only 10 author affiliated institutions have five or more policy-related publications (Fig. 5). Simon Fraser University [13], University of Cambridge [11], University of London [11], Harvard University [9] and University of Ottawa [9] comprise the top five and produce a quarter of all policy research on medical tourism. The bulk of policy research is thus concentrated within a relatively small group of scholars operating out of a small number of academic institutions, working often in mixed-disciplinary teams through interdepartmental collaborations.

### Areas of policy focus

**Fig. 6:**
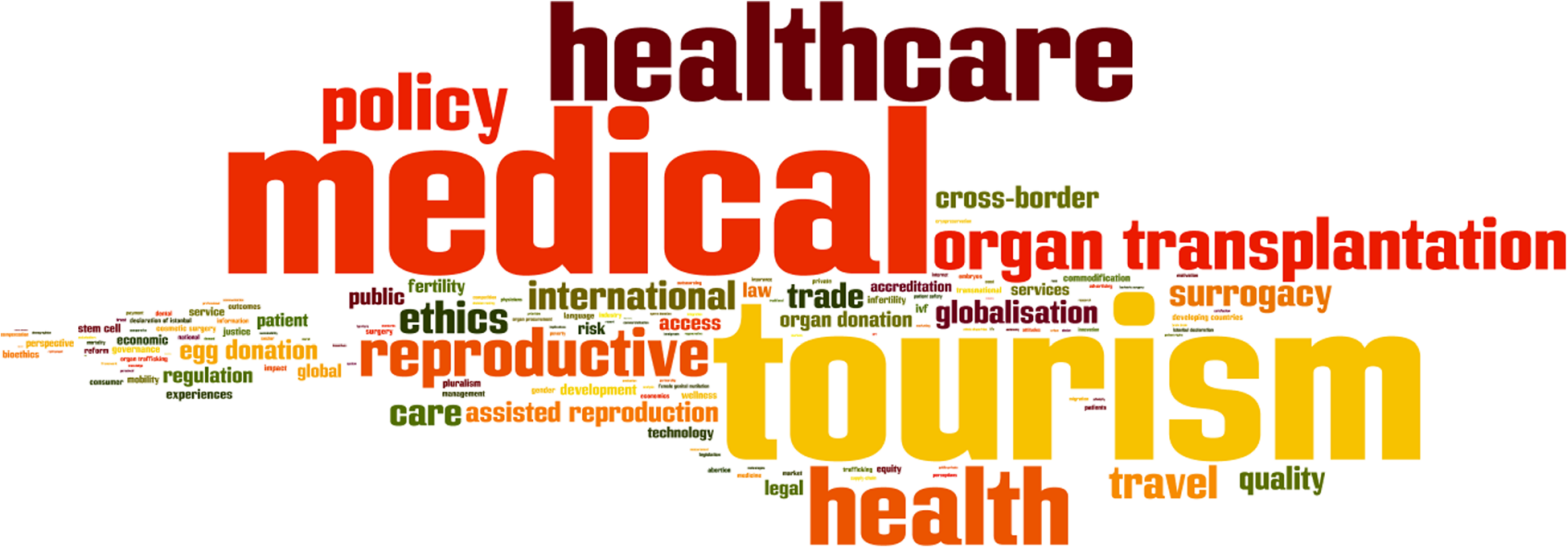
Tag cloud of key themes in policy-related medical tourism research

**Fig. 7:**
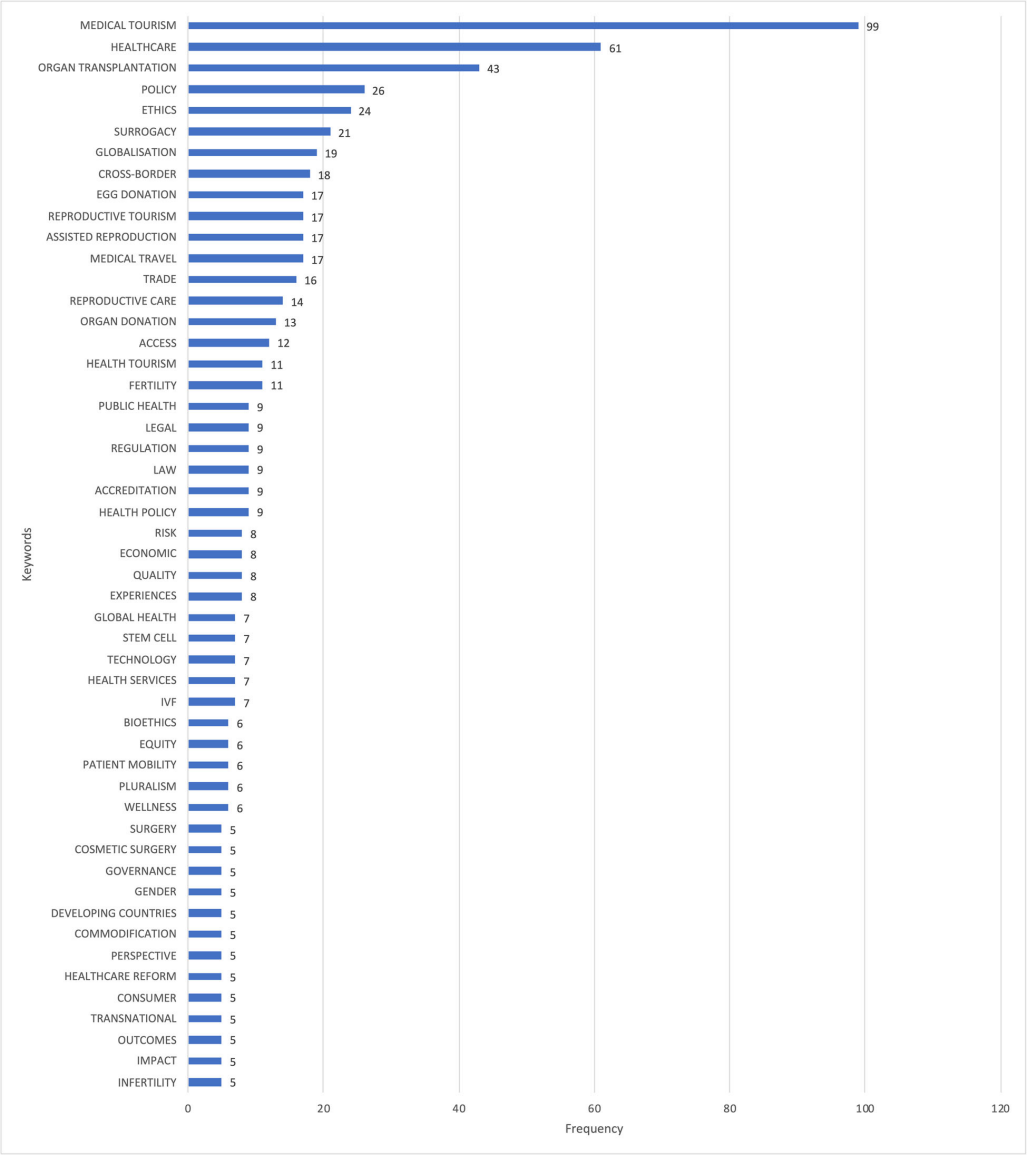
Frequently occurring themes in policy-related medical tourism research

The bulk of policy research has occurred in two areas (Fig. 6 & Fig. 7). The first covers organ transplantation, commercial organ donation, trade and trafficking in solid human organs such as kidneys and liver, and new technologies like stem cell treatment. These practices are typically discussed in the context of transplant tourism. The second encompasses reproductive tourism, which includes gamete donation (donation of eggs and sperms), assisted reproductive technologies like in-vitro fertilization and commercial surrogacy arrangements. Literature in both areas has tended to focus on similar cross-cutting themes: ethical concerns, issues of access and inequity, and legal and regulatory responses to address them. In contrast, there is far less research on the governance and organizational challenges of health systems. Research in this area has reviewed hospital accreditation efforts, and highlighted concerns about disparities in healthcare quality in destination countries, cross-border incompatibilities constraining patient mobility, and the impact on health outcomes and costs of transnational travel for elective procedures like cosmetic, bariatric and dental surgeries that are typically not covered under traditional insurance plans or as part of healthcare entitlements in most countries. These lines of enquiry have received little attention.

### Network linkages in policy research

**Fig. 8:**
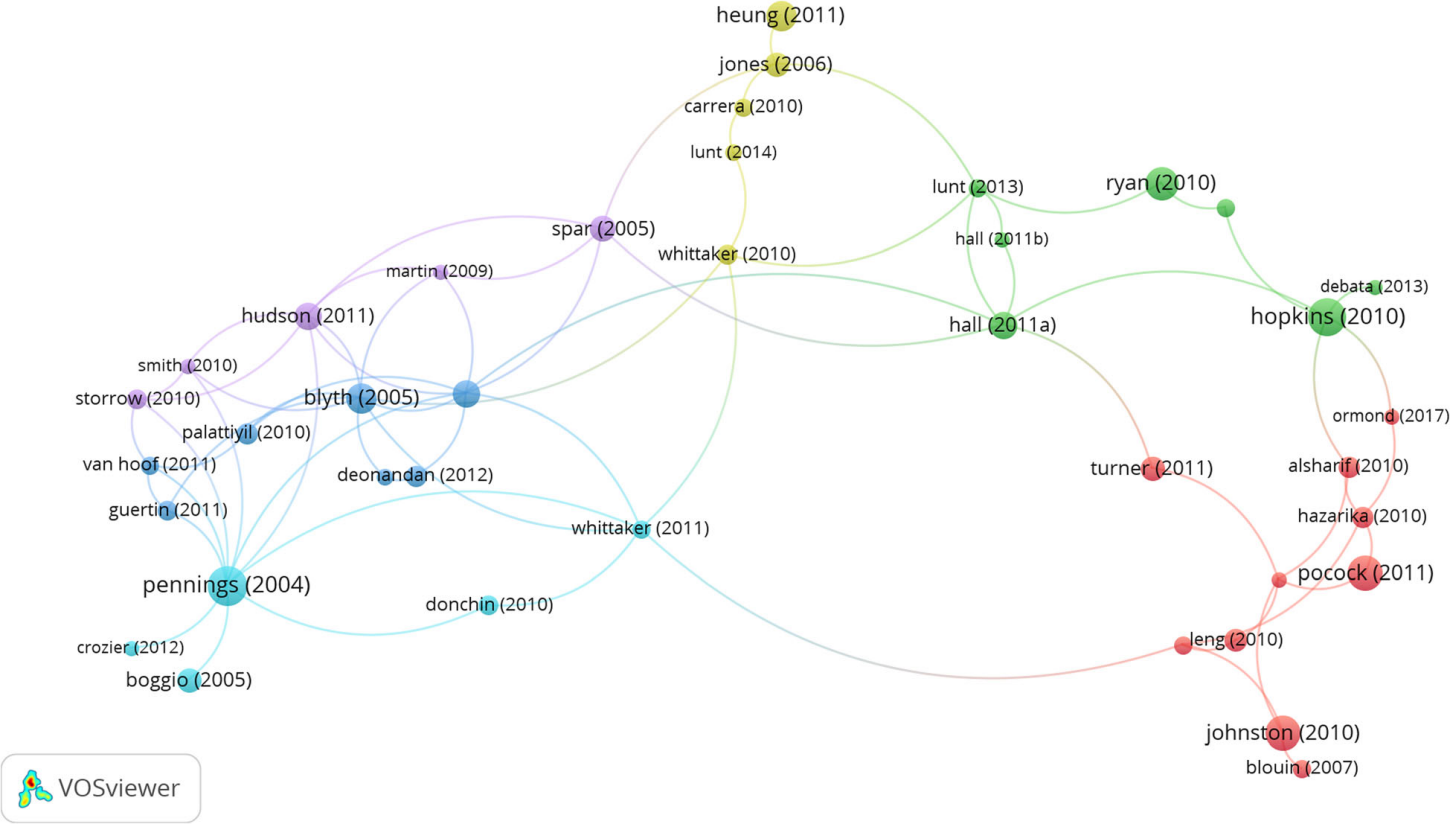
Citation network of policy-related publications on medical tourisms. *Note:* The figure shows how frequently publications (with at least 15 citations) cite or get cited by others in the network. Nodes depict publications and their linkages convey citation relationships. Larger nodes indicate publications with more citations. Publications with close citation relationships are clustered together in nodes of the same color

Table 4 gives a snapshot of the most cited policy-related publications and the key issues they have discussed. These papers have in turn spurred others and led to a growing critical mass of policy work (Fig. 8).

**Table 4:** Most cited policy-related publications on medical tourism

Article	Issues discussed	Total citations
Hopkins, Labonté et al. [36]	Benefits and risks of medical tourism	116
Pennings [37]	Legislative harmonization and regulation of reproductive tourism in Europe	114
Pocock and Phua [38]	Implications of medical tourism for health systems in Thailand, Singapore and Malaysia	104
Johnston, Crooks et al. [8]	Regulatory challenges from poor understanding of the effects of medical tourism	97
Ryan, Sanders et al. [39]	Characteristics of patients undergoing stem cell therapies and policy considerations	81
Heung, Kucukusta et al. [40]	Barriers to medical tourism development in Hong Kong and essential policy interventions	72
Blyth and Farrand [41]	Regulation of assisted conception	66
Hudson, Culley et al. [42]	Policy responses to cross-border reproductive care	59
Hall [43]	Effect of regulation on cross-border trade in health services and implications for global public health	58
Chee [44]	Healthcare reforms in Malaysia and Singapore and the role of the state in developing medical tourism	52

**Fig. 9:**
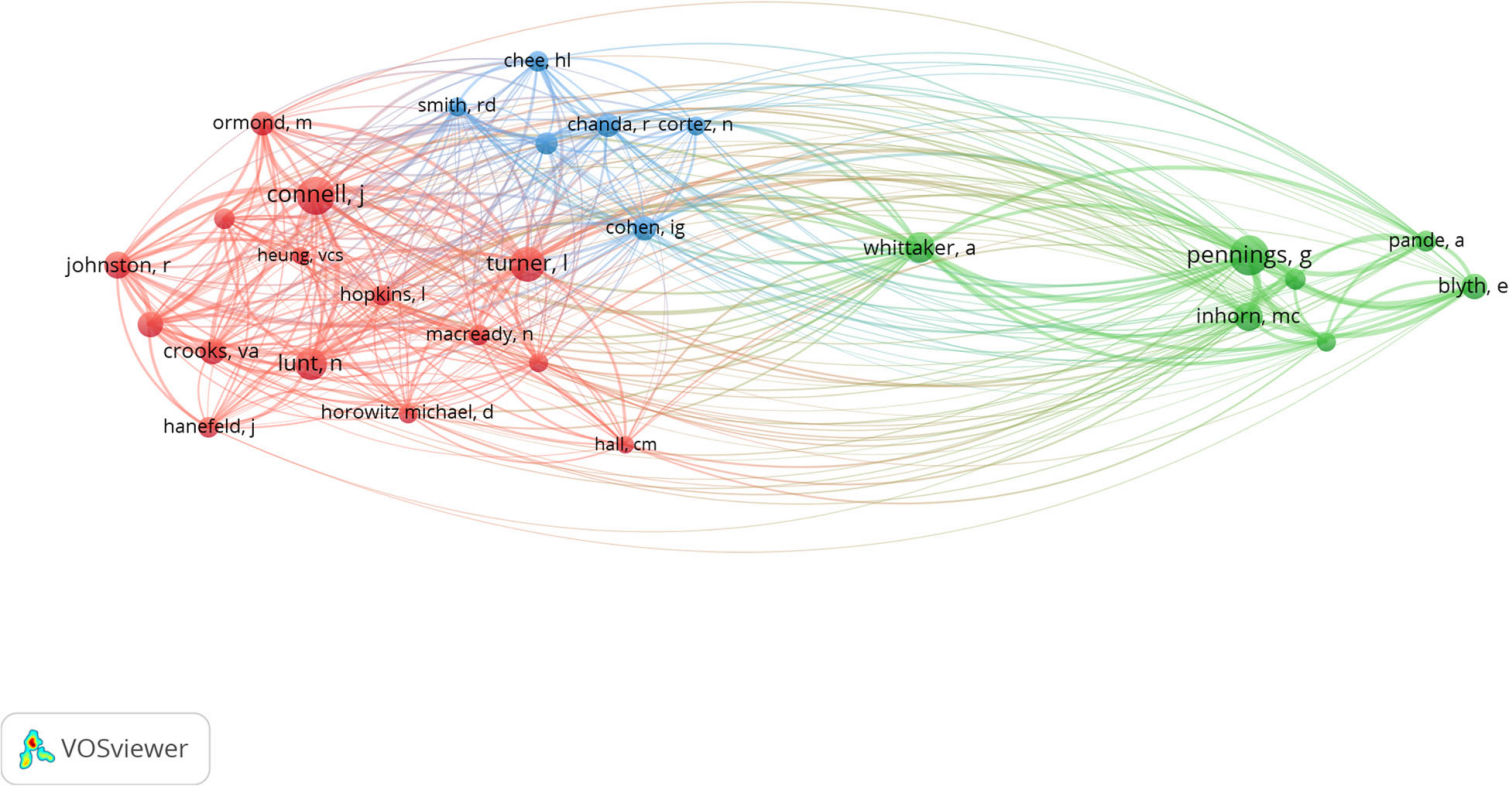
Co-citation network of scholars with policy-related publications on medical tourism. *Note:* The figure shows how frequently scholars (with at least 20 citations) are cited together in publications. Nodes depict authors and their linkages convey co-citation relationships. Larger nodes indicate authors with more citations. Link strengths indicate the frequency with which they are co-cited. Scholars who are co-cited often are clustered together in nodes of the same color

The breadth of scholarship in the field is demarcated by three loose conglomerates of researchers based on their centrality to specific knowledge streams: health systems and policy, economics and regulation of cross-border trade in health services, and reproductive tourism (Fig. 9).

**Fig. 10:**
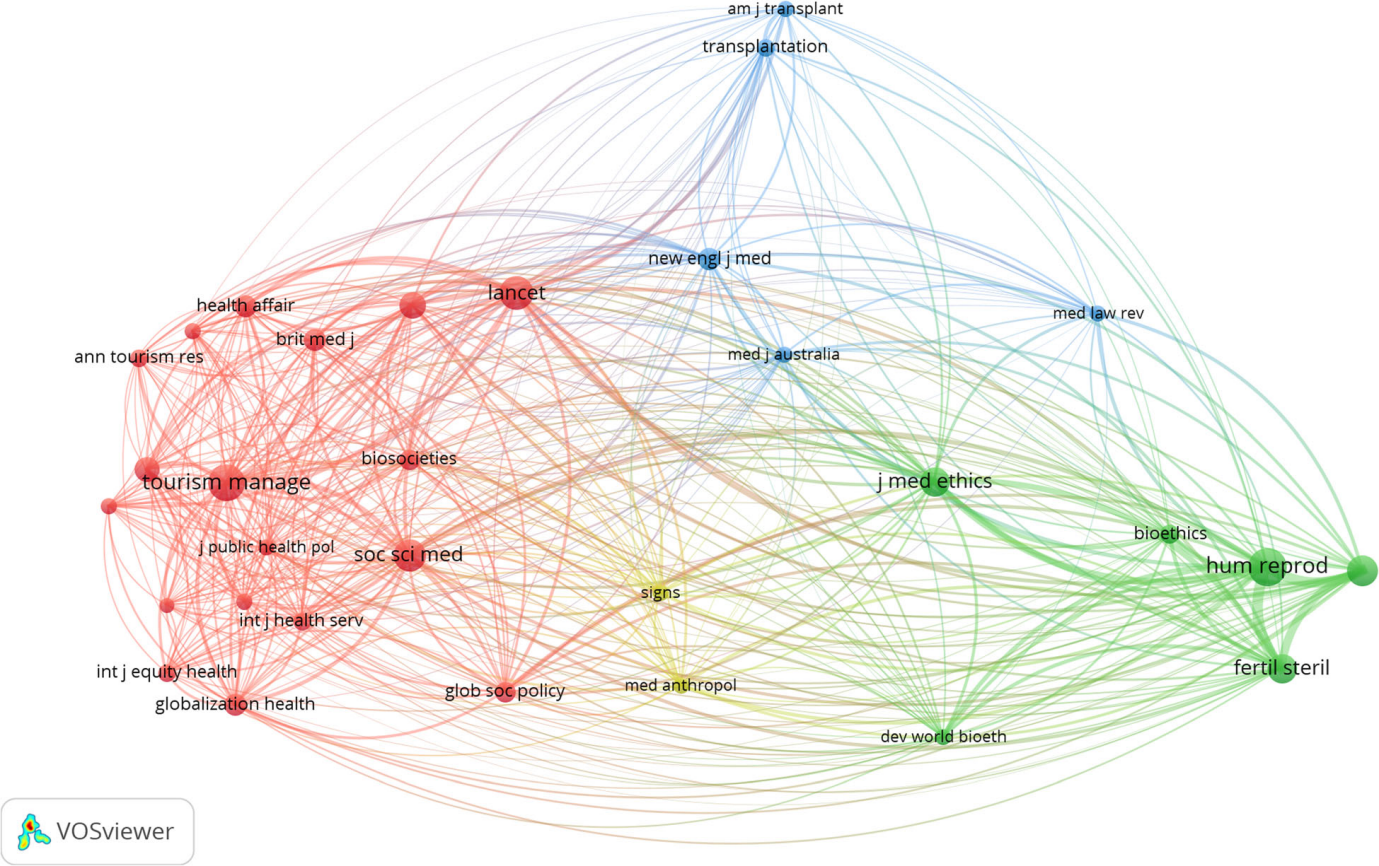
Co-citation network of journals publishing policy research on medical tourism. *Note:* The figure shows how frequently journals (with at least 25 citations) are cited together in publications. Nodes depict journals and their linkages convey co-citation relationships. Larger nodes indicate journals with more citations. Link strengths indicate the frequency with which they are co-cited. Journals that are co-cited often are clustered together in nodes of the same color

As a result, policy-related outputs are spread across different types of journals, depending on the nature of employed paradigms and the object of research. Figure 10 identifies four distinct journal groupings where policy work on medical tourism has appeared: journals in public health, healthcare services and tourism; medical journals; subspecialty journals focused on human reproduction and bioethics; and a small segment of journals that publish sociological and anthropological work. This is interesting not only because it shows how the scholarship is fragmented across a wide disciplinary spectrum, but also because it highlights certain relationships which while not illogical, are not exclusively expected. For instance, ethics is more frequently discussed in the context of reproductive technologies, as compared to medical procedures like organ transplantation. In contrast, research published in tourism journals has been applied to broader issues of global health and health systems development.

### Thematic evolution of policy research

**Fig. 11:**
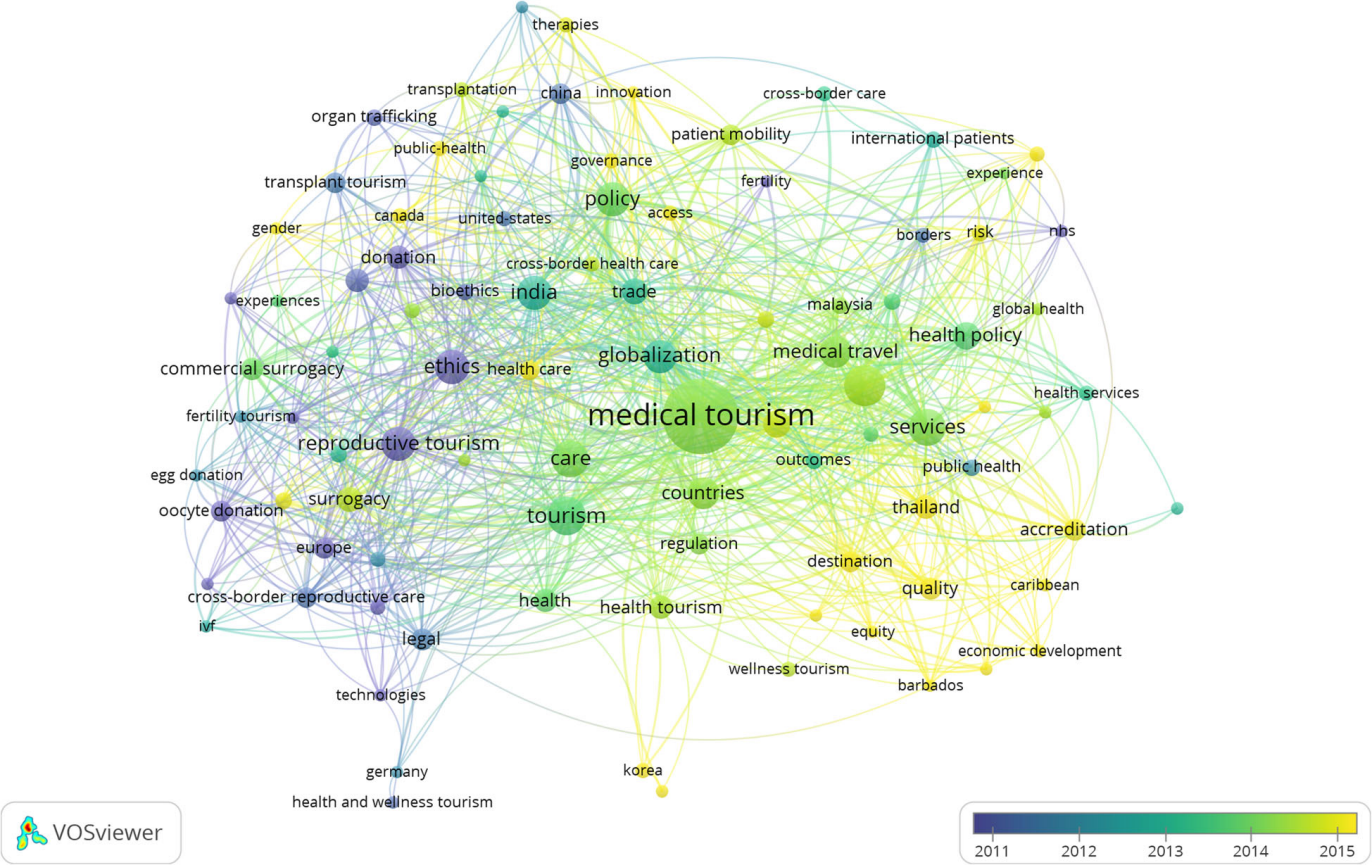
Co-occurrence network of keywords in policy-related publications on medical tourism. *Note:* The figure shows the relatedness of author designated and auto indexed keywords (with at least three occurrences) based on how frequently they occur in the same publications. Nodes depict keywords and their linkages convey co-occurrence relationships. Larger nodes indicate keywords with more occurrences. Link strengths indicate the frequency with which they co-occur. Keywords that co-occur more frequently are clustered together. Nodes are colored based on the frequency with which the keywords occur in publications in different years. Keywords occurring with greater frequency in recent publications are colored lighter than those frequently occurring in the past

While we’ve previously identified the key themes in existing policy research on medical tourism, a network analysis reveals trends that highlight the distinction between *old* and *new* streams of research (Fig. 11 & Table 5). Clusters dealing with reproductive and transplant tourism, and their ethical and legal dimensions (clusters 1–3) represent the older streams which constitute the largest chunk of research, but research in these areas has declined in recent years. Newer clusters (clusters 4–7) have arisen, and new themes of research have emerged within older clusters, reflecting changes in the global policy discourse, the political economy of knowledge production and societal priorities. These include a renewed focus on health systems, particularly issues of regulatory governance, patient safety, cross-border mobility, care quality, accreditation, access, inequity, gender inequality and effects on economic development in destination countries. This indicates a scholarship evolving with transnational shifts in policy agendas, as well as a reorientation towards more action-orientated policy research, just within the span of the current decade.

**Table 5:** Clusters of co-occurring keywords in policy-related publications on medical tourism

Cluster	Keywords
1	assisted reproduction, assisted reproductive technologies, commercial surrogacy, cross-border reproductive care, egg donation, ethics, Europe, exile, fertility tourism, gamete donation, Germany, health and wellness tourism, infertility, IVF, legal, oocyte donation, pluralism, reproductive tourism, reproductive travel, surrogacy, technologies, tourism
2	abroad, cross-border care, developing countries, experience, fertility, global health, globalization, health policy, health services, international patients, medical tourism, medical travel, NHS, outcomes, patient mobility, public health, risk
3	bioethics, China, clinics, cross-border healthcare, donation, governance, healthcare, India, innovation, organ trafficking, organ transplantation, poverty, therapies, trade, transplant tourism, transplantation
4	accreditation, Barbados, Caribbean, countries, destination, economic development, equity, health equity, healthcare, patient safety, quality, regulation, services, Thailand
5	access, Canada, challenges, commodification, experiences, gender, law, legislation, policy, public health, United States
6	borders, impact, international medical travel, Malaysia, perspective, surgery, travel
7	care, health, health tourism, international healthcare, Korea, public policy, wellness tourism

## Discussion

The validity of our findings is limited by the inherent weaknesses of a keyword-based bibliometric analysis. We worked with a fairly narrow and explicit operationalization of policy research, which might have resulted in a number of policy-related works to be overlooked in our search. We focused on published academic research, which might have kept research outputs produced by think tanks, non-governmental agencies, non-profit organizations and governmental departments, outside our purview. These methodological limitations notwithstanding, there are clear trends and patterns in the data, some of which are concerning, others lending some interesting insight.

Only a small proportion of medical tourism research explicitly addresses policy, and few researchers situate their work in the context of contemporary policy debates, or discuss the policy implications of their work. The relative dearth of critical policy focus is not unique to medical tourism research, and is prevalent in other social science related disciplines, such as in housing [45], education [46, 47], human geography [48–50], tourism [13] and water sector studies [51]. This is likely because the study of *policy* as a necessary applied component of social science research and its practical significance for the redress of pressing societal concerns are less well-recognized in academic research. This is a potential issue, because even if a significant body of research is implicitly policy relevant, failure to make the policy connection is a lost opportunity to frame the public debate and influence policy thinking on medical tourism. For example, while research on medical tourism in Canada has highlighted the ethical, medicolegal and operational challenges arising from Canadian patients traveling abroad to seek medical treatment, it does not adequately address the domestic policy-level drivers that compel them to travel overseas, or gaps in the legal regulation of cross-border healthcare markets that give rise to or exacerbate these challenges [4]. Nonetheless, the quantum of policy-oriented research in the field has been steadily increasing as public policy and the policy sciences have gained greater traction as distinct academic disciplines, and scholars and policymakers have become more interested in understanding how globalization processes, transnational and domestic governance regimes, and government priorities and actions influence the dynamics of international medical travel, and its effects.

Moreover, while research on medical tourism shows much interdisciplinarity, there is relatively little conceptual and theoretical crossover between modern scholarship in the policy sciences, and the various disciplinary perspectives in medical tourism research. Public policy concepts or frameworks are occasionally applied, and when applied, their treatment is often superficial or incidental. This is possibly due to the fact that the bulk of medical tourism research has been conducted by researchers from *non-policy* backgrounds such as bioethics, public health, reproductive health, transplant medicine, economics, sociology, geography and tourism studies, with their own distinct disciplinary foundations, and little theoretical grounding in public policy. Conversely, few political scientists and public policy and public administration scholars have researched this field. As a result, little knowledge diffusion has occurred in this regard.

Having said that, there have been attempts to draw on and engage with policy theory, such as Whittaker’s [10] framework for conceptualizing the equity effects of medical tourism in low- and middle-income countries, He, Lai, and Ching’s [52] use of *policy adaptation* to explain changes in organ transplantation policies in Asia, and Bochaton’s [53] examination of medical tourism development in Thailand through paradigms of *transnational assemblage* and *national therapeutic landscapes*. Whittaker’s [10] work, for example, draws attention to the interplay of demand side and supply-side factors and local policy settings which produces inequitable adverse effects on health human resources and access to health systems in destination countries, leading her to advocate for cross-sectoral policy coherence, and policy responses to limit the need for citizens from source countries to travel overseas for medical treatment unless critical. He, Lai, and Ching’s [52] article analyses adaptability in organ transplantation policies in ten Asian countries to demonstrate how countries that have progressively liberalized their policy regimes to expand donor eligibility and legalize compensation have been more effective in balancing ethical concerns with evolving domestic demands. Bochaton [53] applies sociological constructs developed by Wilson [54] and Ormond [55] in the context of transnational medical travel to explain how changing political and economic conditions in Thailand aided by government action created a uniquely suitable environment to attract medical tourists from neighbouring countries like Laos. Though such instances are infrequent, these articles are fine examples of how policy-oriented theoretical frameworks can provide researchers potential lenses through which to assess the nature of policy problems associated with medical tourism in both source and destination countries and analyze the contextual efficacy of policy solutions.

While policy research tends to diffuse more rapidly on average, the most influential policy-related publications on medical tourism are relatively less so as compared to popular non-policy publications on the subject. Disparities in the geographical distribution of policy research production are intriguing, with some countries displaying a proportionately higher proclivity to produce policy-focused research than others, possibly reflecting the strength of their policy faculty, higher levels of resource allocation to policy-focused departments, research centres and think-tanks, emphasis on research projects to demonstrate societal impact for securing grant funding, and the culture of research-driven governance and policymaking. Likewise, policy research on medical tourism is more prevalent in certain disciplines and tends to appear more frequently in certain classes of journals. For instance, policy publications are concentrated in interdisciplinary health and social policy journals, while field-specialized journals that cater to practitioners publish less policy-oriented work. Only a small proportion of overall policy research in this field is published in journals in political science and public administration, which corroborates Hall and Jenkins’ [13] concerns about the relative lack of theoretical engagement by scholars in public policy and the policy sciences, which we alluded to earlier.

Policy research on the subject is concentrated within a relatively small group of scholars who are self-organized into distinct networks of research communities. This has led to the emergence of separate strands of policy scholarship. Consequently, while there is much diversity in policy ideas, certain policy areas such as reproductive and transplant tourism have traditionally received far more attention by researchers, and have hegemonized the policy space to the neglect of more holistic governance and health system considerations, though the latter have seen some resurgence of late. The emphasis in policy research in the last decade has shifted to address newer themes, particularly health system impacts of medical tourism, and regulatory fitness to address its myriad policy challenges. Recent policy articles, for instance, have drawn on existing literature and empirical studies in the field to examine how healthcare reforms in various countries have influenced cross-border flows of medical tourists, and how medical travel has in turn impacted national health systems in those countries [38, 56, 57]. Béland and Zarzeczny [56], for example, have explored the nexus between medical tourism in the US and Canada and the characteristics of their healthcare systems, and suggested how adopting a comparative institutionalist research perspective can provide policymakers with insights into the nature and significance of this relationship for healthcare access and other policy objectives. Likewise, Pocock and Phua [38] have shown how medical tourism has exacerbated existing public-private inequities in healthcare, and which regulatory measures have worked or failed in addressing them, based on case study evidence from Thailand, Singapore and Malaysia. Bustamante [57] has examined the implications of bi-national health insurance policy reforms in the US and Mexico for healthcare coverage to vulnerable populations in both countries, and the political and legal challenges which make them difficult to accomplish on a large scale. Such works offer practical evaluative frameworks to policymakers through which to examine the drivers, dynamics and challenges of medical tourism, and assess the efficacy and implications of current or proposed policy interventions, so that more effective regulatory solutions can be designed. While these represent only a segment of the vast canvas of policy research, their emergence reflects the changing policy discourse and research priorities on medical tourism towards more problem-centred and action-oriented research, and is indicative of the kind of policy work that more of is required to move the research agenda forward and address its policy challenges. This shift mirrors the broader movement within the policy sciences itself, away from theoretical conceptualization of policy problems and governance regimes towards technical policy analysis and governance design aspects [29].

## Conclusion

In this paper, we scoped the academic literature to ascertain where is the policy in medical tourism research. Our first objective was to determine how much of medical tourism research is explicitly policy-oriented. Our second objective was to outline the contours of policy research on medical tourism and ascertain what aspects of policy are being studied or neglected. To clarify, this paper reviews not the state of policy on medical tourism, but the state of policy in research on medical tourism. Overall, we find that discussion on policy in medical tourism research is relatively limited, and that what discussion there is, is fragmented and lopsidedly inclined towards select policy areas.

Moving forward, there is need for greater engagement by policy scholars on the subject, and for medical tourism researchers to more explicitly consider how their research might contribute to the understanding and resolution of the policy challenges of medical tourism. While healthcare is one policy field with obvious policy connotations, medical tourism spans several other policy arenas, with distinct problem perspectives and solution parameters. These need to be recognized and adequately reflected in policy research.

## Data Availability

The datasets used and/or analysed for the current study are available from the corresponding author on reasonable request.

## Abbreviations

EU: European Union
NHS: National Health Service
PRISMA: Preferred reporting Items for Systematic review and Meta-Analyses
UK: United Kingdom
US: United States
WoS: Web of Science
